# Association of dietary patterns and endoscopic gastric mucosal atrophy in an adult Chinese population

**DOI:** 10.1038/s41598-019-52951-7

**Published:** 2019-11-12

**Authors:** Song Lin, Tao Gao, Chongxiu Sun, Mengru Jia, Chengxia Liu, Xingbin Ma, Aiguo Ma

**Affiliations:** 1 The College of Public Health, Qingdao University, 38 Dengzhou Road, Qingdao, Shandong 266021 China; 2 Digestive endoscopy center, Hospital Affiliated Binzhou Medical University, Binzhou, 256603 China

**Keywords:** Gastritis, Risk factors

## Abstract

Atrophy gastritis harbor a high risk for the development of dysplasia and gastric cancer. The study investigated the relationships of specific dietary patterns and endoscopic gastric mucosal atrophy. In this cross-sectional study, we enrolled 574 consecutive outpatients who were diagnosed as chronic gastritis according to endoscopic examination. Dietary intakes of study individuals was assessed using the semi-quantitative food group frequency questionnaire. Logistic regression analyses were used to evaluate the relationship between dietary patterns and endoscopic gastric mucosal atrophy adjusted for potential confounders. A total of 574 participants were included, 286 with endoscopic gastric mucosal atrophy. Three dietary patterns were identified by factor analysis. “Alcohol and fish” (tertile 1 vs. tertile 3: adjusted odds ratio = 1.85, 95% confidence interval: 1.06–3.22) and “coarse cereals” (tertile 1 vs. tertile 3: adjusted odds ratio = 2.05, 95% confidence interval: 1.24–3.39) were associated with an increased risk for endoscopic gastric mucosal atrophy but a “traditional” pattern was not. Dietary pattern was not associated with gastric mucosal atrophy in women or in participants with *H. pylori* infection. A high adherence to both “Alcohol and Fish” and “Coarse cereals” dietary patterns seem to be associated with higher odds of endoscopic gastric mucosal atrophy in men and in patients without *H. pylori* infection. Further prospective cohort studies needed to confirm these findings.

## Introduction

Gastric mucosal atrophy is a common, age-related multifactorial disease associated with *Helicobacter pylori* infection and diet^1–3^. It is estimated that as many as 25% of people worldwide are at risk of loss or atrophy of normal gastric mucosal glands^4^. The prevalence of chronic atrophy gastritis varies regionally, from 25.8% in China^5^ to 40.7% in Korea^6^. Gastric mucosal atrophy is a precursor lesion in the development of gastric carcinoma^7,8^. The overall incidence of gastric cancer has been declining in Asian countries including China^9^, but most people infected by *H. pylori* remain asymptomatic for life despite evidence of chronic gastritis^10^. *H. pylori* infection alone might not be sufficient to cause gastric cancer even though the World Health Organization considers it to be a class-I carcinogen. Diet may also play a key role in gastric carcinogenesis. For example, some studies have found that a high-salt diet was independently associated with gastric carcinoma and that Western diets rich in salt, meat, animal fat, starchy food and alcohol increase the risk of precancerous lesions or gastric cancer^11–17^. Diets rich in fruits and vegetables appear to reduce gastric cancer risk^18,19^. Completion of the sequence of events that occur in the gastric mucosa before the manifestation of gastric cancer may take decades^8^. Most studies of the role of diet have focused on gastric cancer, but increased awareness of the association of dietary factors with development of precancerous lesions would help in the formulation of targeted prevention.

Rather than focusing on individual nutrients or foods, dietary pattern analysis might be the most feasible method to assess the relationship between diet and disease and to help nutritionists to provide dietary recommendations in public health practice^20^. The characterization of diet patterns by factor analysis has been proven to predict the risk of coronary heart disease^21^, colorectal cancer^22^, and gallbladder disease^23^. To our knowledge, the relationship of dietary factors and endoscopic gastric mucosal atrophy has not been well established. The study aim was to identify the role of dietary patterns and risk factors associated with gastritis and of potential help in the prevention of gastric carcinoma.

## Methods

### Study population

**Figure 1:**
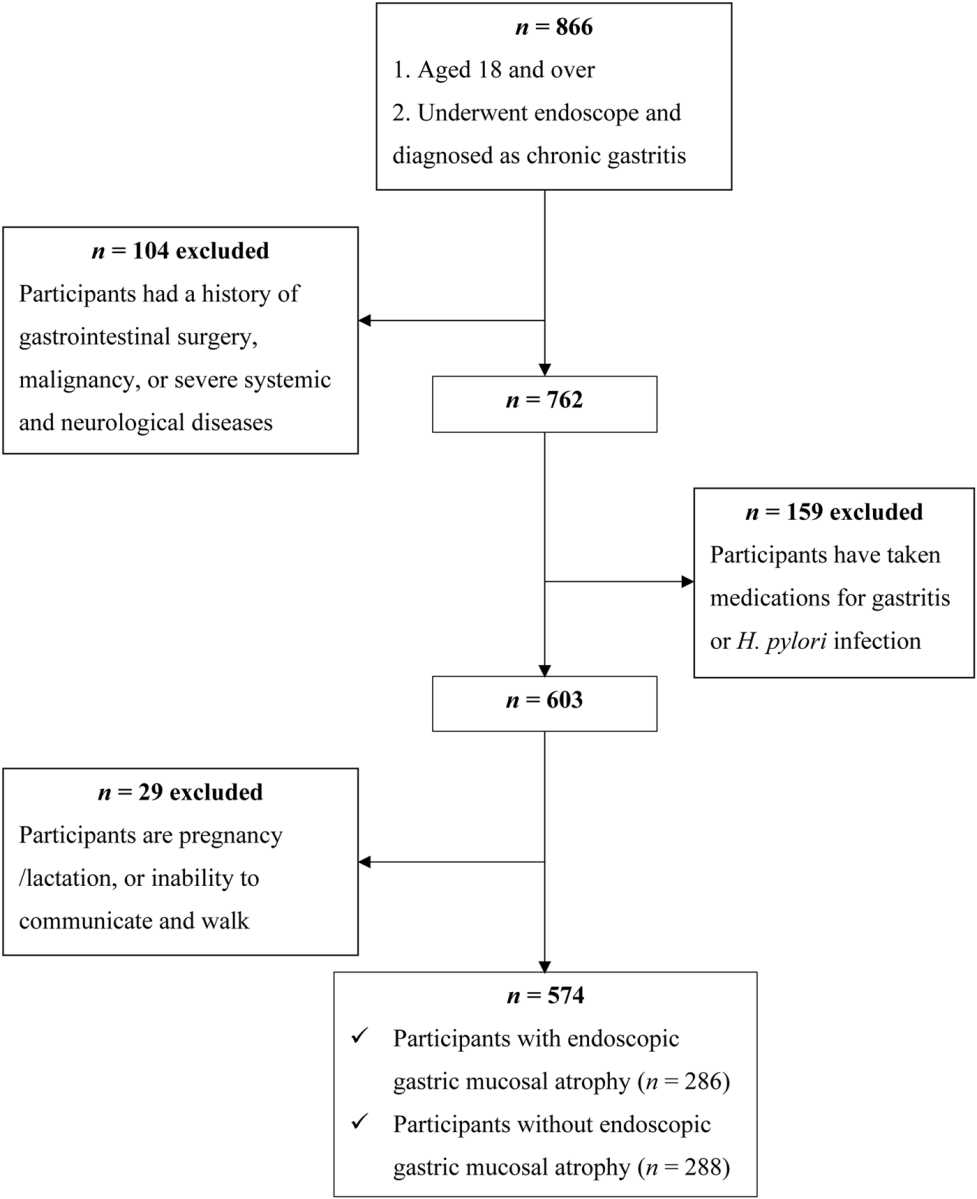
Flow chart of the screening process of the selection of eligible participants.

This cross-sectional study investigated the relationship of diet and endoscopic gastric mucosal atrophy in a Han Chinese population in Binzhou city Shandong Province, China. The participants were recruited between April and October 2018 at the Outpatient Digestive Endoscopy Center at the Binzhou Medical University Affiliated Hospital, which is the only tertiary hospital in the region. Patients ≥18 years of age with a endoscopic diagnosis of chronic gastritis were eligible for inclusion. Patients with a history of gastrointestinal surgery, malignancy, or severe systemic of neurological disease, a history of gastritis or *H*. *pylori* infection, pregnancy or at lactation, or unable to communicate or walk normally were excluded. A total of 574 consecutive outpatients who met the above eligibility were included in the analyses (Fig. 1). The study was approved by the ethics committee of Binzhou Medical University Hospital and conducted in line with the ethical guidelines of Declaration of Helsinki. Informed consent was obtained from all participants.

### Assessment of dietary intake

Dietary intake during the previous year was assessed with a semiquantitative food group frequency questionnaire (FGFQ) administered by medical staff and study investigators during face-to-face interviews. The questionnaire included items on the frequency (“three times a day”, “twice a day”, “once a day”, “two to three times a week”, “once a week”, “once every two weeks”, “once a month”, or “never”) and portion size in grams of 23 food group items. The daily intake in grams was calculated by multiplying the portion size of each food group item by the daily consumption frequency (3, 2, 1, 0.357, 0.143, 0.071, 0.033, or 0). The patients were asked to recall consumption of food groups rather than nutrients during the previous year because of the short 20-minute interval between administration of the spray oral anesthetic and the endoscopy procedure. Commonly consumed food groups and recipes were chosen by a nutritionist (Supplementary Table 1). Only 180 individuals completed both FGFQ and a 24-h dietary recall. The Spearman rank correlations are shown in Supplementary Table 2. Total energy intake (kcal/day) was calculated by NutriSurvey (http://www.nutrisurvey.de) using the 24 h recall data.

### Diagnosis of chronic atrophic gastritis and H. pylori infection

Endoscopies were performed and evaluated by experienced practitioners. Gastric mucosal atrophy was described and graded by the Chinese Society of Digestive Endoscopy criteria^24^. A rapid urease test was used to diagnose *H. pylori* infection^25^.

### Participant characteristics

Potential confounding social and demographic variables, including age, sex, height, weight, body mass index (BMI, kg/m^2^), education, place of residence, occupation, income, smoking status, drinking status, and histories of diabetes, hypertension, and anticoagulant use were collected with a structured questionnaire.

### Statistical analysis

Statistical analysis was performed with SPSS 25.0 (IBM Corp., Armonk, NY, USA) and Stata version 15.1 (Stata Corporation, College Station, TX, USA). All *p*-values were two-sided. Dietary patterns were analyzed by principle component analysis (PCA) with varimax rotation. The number of patterns was determined by the cumulative contribution rate of variance, eigenvalue >1.45, and the interpretability of the results. Food groups with a rotated factor loading of >|0.4| were considered the primary components of each pattern. Dietary pattern scores were calculated by summing the products of the standardized intakes of each food group multiplied by regression coefficients and were reported as tertiles. The values of continuous variables were reported as means and standard deviations. Categorical variables were reported as numbers and percentages. Between-group differences in participants with or without endoscopic gastric mucosal atrophy were compared by Student’s *t*-tests. Chi-square tests were used to compare percentage differences of categorical variables in participants with or without endoscopic gastric mucosal atrophy. Differences in patient characteristics across dietary pattern scores were evaluated by chi-square tests for categorical variables and the analysis of variance for continuous variables. The association of adherence to each dietary pattern and the odds of endoscopic gastric mucosal atrophy was tested by binary logistic regression. Model 1 was adjusted for age and sex. Model 2 included BMI, education (<6, 6–12, or >12 years), residence (urban or rural), marital status (single, divorced, widowed, or married), occupation (supervisory, company employee, laborer, self-employed, student, farmer, or retried) income (<1000, 1000–3000, >3000 CNY/month/individual), smoking (yes or no), drinking (yes or no). Model 3 was further adjusted for *H*. *pylori* infection (yes or no), history of diabetes (yes or no), history of hypertension (yes or no), history of anticoagulant use (yes or no), and total energy intake. Assuming that total energy intake was missing in randomly distributed participants, missing data were compensated by multivariate imputation of chained equations^26,27^. Twenty imputed datasets were created to reduce sampling variability and the estimates were combined based on Rubin’s rules^28^.

## Results

The characteristics of the 574 participants are shown in Table 1. The point prevalence of endoscopic gastric mucosal atrophy was 49.8%. Participants with or without endoscopic gastric mucosal atrophy showed significant differences in terms of marital status (*p* = 0.044), occupation (*p* = 0.001), *H. pylori* infection status (*p* < 0.001), history of hypertension (*p* = 0.001), history of taking anticoagulants (*p* = 0.015). Compared with those without endoscopic gastric mucosal atrophy, participants with gastric mucosal atrophy showed older age (*p* < 0.001), lower education (*p* < 0.001), and higher income (*p* = 0.012).

**Table 1:** General characteristics of participants by having or not having endoscopic gastric mucosal atrophy.

Variables^a^	Participants with endoscopic gastric mucosal atrophy n = 286 (49.8%)	Participants without endoscopic gastric mucosal atrophy n = 288 (50.2%)	*P* ^b^
**Gender**			0.551
Male	162 (56.6%)	156 (54.2%)	
Female	124 (43.4%)	132 (45.8%)	
**Age group, year**			<0.001
18–44	52 (18.2%)	154 (53.5%)	
45–54	109 (38.1%)	88 (30.6%)	
≥55	125 (43.7%)	46 (16.0%)	
BMI, kg/m^b^	24.1 ± 3.46	23.4 ± 3.64	0.020
**Residence**			0.606
Urban	84 (29.4%)	79 (27.4%)	
Rural	202 (70.6%)	209 (72.6%)	
**Year of education, year**			<0.001
<6	80 (28.0%)	54 (18.8%)	
6–12	171 (59.8%)	165 (57.3%)	
>12	35 (12.2%)	69 (24.0%)	
**Marital status**			
Single/Divorced/Widowed	4 (1.4%)	12 (4.2%)	0.044
Married	282 (99.6%)	276 (95.8%)	
**Occupation**			0.001
Authority unit	39 (13.6%)	52 (18.1%)	
Company employee	12 (4.2%)	18 (6.3%)	
Worker	39 (13.6%)	46 (16.0%)	
Self-employer	41 (14.3%)	61 (21.2%)	
Student	2 (0.7%)	10 (3.5%)	
Farmer	141 (49.3%)	93 (32.3%)	
Retried	12 (4.2%)	8 (2.8%)	
**Income, CNY/month/per-individual**			0.007
<1000	67 (23.4%)	57 (19.8%)	
1001–3000	110 (38.5%)	84 (29.2%)	
>3000	109 (38.1%)	147 (51.0%)	
**Smoking**			0.455
Yes	133 (46.5%)	125 (43.4%)	
No	153 (53.5%)	163 (56.6%)	
**Drinking**			0.474
Yes	78 (27.3%)	71 (24.7%)	
No	208 (72.7%)	217 (75.3%)	
***H. pylori*** **infection**			<0.001
Yes	109 (38.4%)	55 (19.2%)	
No	175 (61.6%)	231 (80.8%)	
**History of diabetes**			
Yes	19 (6.6%)	13 (4.5%)	0.266
No	267 (93.4%)	275 (95.5%)	
**History of hypertension**			0.001
Yes	49 (17.1%)	22 (7.6%)	
No	237 (82.9%)	266 (92.4%)	
**History of taking anticoagulants**			0.015
Yes	22 (7.7%)	9 (3.1%)	
No	264 (92.3%)	279 (96.9%)	
**Total energy intake, kcal/day** ^**c**^	1804.3 ± 719.7	1830.8 ± 706.1	0.868

Abbreviations: BMI, body mass index; CNY, Chinese yuan.

^a^Values presented as percentage for categorical variables and mean ± SD for continuous variables.

^b^Chi-square test was used to compare the percentage between participants with or without endoscopic gastric mucosal atrophy. Student’s t-test analyses was used to compare the mean values between participants with or without endoscopic gastric mucosal atrophy.

^c^From 180 individuals (89 participants with endoscopic gastric mucosal atrophy, 91 participants without endoscopic gastric mucosal atrophy).

### Extraction of dietary patterns

The PCA analysis yielded three independent diet patterns that explained 24.5% of the variation in food consumption (Table 2). The first was “alcohol and fish,” which included high consumption of wine, beer, fresh water and sea fish. The second was “traditional,” with high consumption of vegetables, wheat products and red meat, and the third was “coarse cereals,” with high consumption of whole grain cereals, legumes, and poultry. The participant characteristics and dietary pattern tertiles are shown in Table 3. Participants with an “alcohol and fish” pattern were more likely to be men, smokers, drinkers, supervisory or company employees, urban residents with high incomes and education levels, and histories of hypertension or anticoagulant use. Those adhering to a “traditional” pattern were more likely to be men, younger, smokers, drinkers, have high incomes, low BMI, and low education level. Those adhering to the “coarse cereals” pattern were more likely to be men, younger, supervisory employees, and urban residents with high education and income levels.

**Table 2:** Principal component analysis of 23 food groups.

Food groups	Alcohol and fish	Traditional	Coarse cereals
Fresh water fish	0.74	—	—
Sea fish	0.73	—	—
Wine	0.68	—	—
Beer	0.65	—	—
Legumes	—	—	0.41
Tea	—	—	—
Poultry	—	—	0.46
Red meat	—	0.50	—
Sugar-sweetened beverage	—	—	—
Potatoes	—	—	—
Vegetables	—	0.73	—
Fruits	—	—	—
Lard oil	—	—	—
Egg	—	—	—
Salted vegetables	—	—	—
Soya-bean oil	——	—	—
Coarse cereals	—	—	0.71
Dairy and its product	—	—	—
Cooked wheaten food	—	0.72	—
Processed meat	—	—	—
Corn oil	—	—	—
Peanut oil	—	—	—
Cooked rice food	—	—	—
Percent of variance explained (%)	10.30%	7.27%	6.88%

Factor loading > 0.4 are listed.

**Table 3:** General characteristics of the study participants cross categories of dietary patterns scores.

Variables^a^	Alcohol and fish			*p* ^b^	Traditional			*p* ^b^	Coarse cereals			*p* ^b^
	T1 (191)	T2 (190)	T3 (193)		T1 (191)	T2 (190)	T3 (193)		T1 (191)	T2 (190)	T3 (193)	
**Gender, males**	39.8	48.4	77.0	<0.001	34.7	51.0	80.2	<0.001	57.1	46.8	62.2	0.009
**Age group**				0.372				0.011				0.001
18–44	30.9	35.8	40.9		27.4	37.5	42.7		29.3	32.1	46.1	
45–54	37.2	34.7	31.1		34.7	35.4	32.8		39.3	40.5	23.3	
≥55	31.9	29.5	28.0		37.9	27.1	24.5		31.4	27.4	30.6	
**BMI**	23.5 ± 3.7	23.5 ± 3.5	24.2 ± 3.5	0.056	23.9 ± 3.7	23.1 ± 3.3	24.2 ± 3.6	0.012	23.8 ± 3.5	23.4 ± 3.5	24.0 ± 3.7	0.328
**Residence**				<0.001				0.596				0.033
Urban	20.9	23.7	40.4		31.1	26.6	27.6		24.1	25.8	35.2	
Rural	79.1	76.3	59.6		68.9	73.4	72.4		75.9	74.2	64.8	
**Year of education**	<0.001				0.03				<0.001			
<6	29.8	23.7	16.6		30.5	22.9	16.7		31.4	21.6	17.1	
6–12	59.2	61.1	55.4		51.6	59.4	64.6		56.5	64.7	54.4	
>12	11.0	15.3	28.0		17.9	17.7	18.8		12.0	13.7	28.5	
**Marital status**				0.783				0.931				0.144
Single/Divorced/Widowed	3.1	2.1	3.1		3.2	2.6	2.6		1.6	2.1	4.7	
Married	96.9	97.9	96.9		96.8	97.4	97.4		98.4	97.9	95.3	
**Occupation**				<0.001				0.718				0.001
Authority unit	11.5	10.5	25.4		15.3	14.6	17.7		9.4	14.2	23.8	
Company employee	1.6	6.8	7.3		4.7	5.2	5.7		5.8	5.8	4.1	
Worker	14.1	17.4	13.0		15.3	15.6	13.5		15.2	12.6	16.6	
Self-employer	11.0	22.1	20.2		14.2	17.2	21.9		16.8	17.4	19.2	
Student	3.7	1.1	1.6		2.1	3.1	1.0		0.5	3.2	2.6	
Farmer	53.4	39.5	29.5		43.7	41.7	37.0		50.8	42.6	29.0	
Retried	4.7	2.6	3.1		4.7	2.6	3.1		1.6	4.2	4.7	
**Income, CNY/month/per-individual**	<0.001				<0.001				0.001			
<1000	31.9	21.6	11.4		34.2	20.3	10.4		26.2	19.5	19.2	
1001–3000	36.1	37.9	27.5		27.4	39.1	34.9		37.7	40.0	23.8	
>3000	31.9	40.5	61.1		38.4	40.6	54.7		36.1	40.5	57.0	
**Smoking, yes**	38.7	36.8	59.1	<0.001	36.3	43.8	54.7	0.001	41.9	41.1	51.8	0.062
**Drinking, yes**	6.8	13.7	57.0	<0.001	11.6	21.9	44.3	<0.001	27.2	20.5	30.1	0.093
***H. pylori*** **infection, yes**	30.9	23.9	31.4	0.201	27.1	25.1	34.0	0.131	24.2	27.1	34.9	0.058
**History of diabetes, yes**	6.8	7.4	2.6	0.083	7.9	6.3	2.6	0.07	5.2	4.7	6.7	0.674
**History of hypertension, yes**	7.9	13.7	15.5	0.058	16.8	9.9	10.4	0.072	11.0	11.1	15.0	0.388
**History of taking anticoagulants, yes**	3.7	4.2	8.3	0.09	5.3	4.7	6.3	0.791	7.3	4.2	4.7	0.346

Abbreviations: BMI, body mass index; CNY, Chinese yuan.

^a^Values presented as percentage for categorical variables and mean ± SD for continuous variables.

^b^Chi-square test for categorical variables and the ANOVA analyses for continuous variables.

After controlling for potential confounders, participants who adhered to the “alcohol and fish” pattern were more likely to be drinkers, non-farmers, without a history of hypertension. Those who adhered to the “traditional” pattern were more likely to be men, younger than 50 years of age, and drinkers. Those who adhered to the “coarse cereals” pattern were more likely to be smokers, have an *H*. *pylori* infection, and have a high education level (Supplementary Table 3).

### Association between dietary patterns and endoscopic gastric mucosal atrophy

Table 4 shows the effect of adherence to the dietary patterns on the risk of endoscopic gastric mucosal atrophy. Model 1 found that the “alcohol and fish” and “coarse cereals” patterns were both positively associated with endoscopic gastric mucosal atrophy but that the “traditional” pattern was not. After controlling for potential confounders, adherence to the “alcohol and fish” dietary pattern was related to an increased prevalence of endoscopic gastric mucosal atrophy, but the association was significant only for the highest tertile (tertile 1 vs. tertile 3 adjusted OR = 1.84, 95% CI: 1.06–3.20, *p*-value for trend = 0.040). Adherence to the “coarse cereals” pattern also increased the risk of endoscopic gastric mucosal atrophy (tertile 1 vs. tertile 3: adjusted OR = 2.04, 95% CI: 1.24–3.38, *p*-value for trend = 0.005). Adherence to the “traditional” pattern was not found to influence the risk of endoscopic gastric mucosal atrophy.

**Table 4:** Multivariable-adjusted odds ratios for endoscopic gastric mucosal atrophy across tertiles of dietary patterns’ scores.

	Cases/non-cases^a^	Model 1	*p* for trend	Model 2	*p* for trend	Model 3	*p* for trend
**Alcohol and fish**			0.02		0.041		0.040
Tertile 1 (low)	91/100	Reference		Reference		Reference	
Tertile 2	87/103	0.95 (0.61–1.48)		0.98 (0.62–1.56)		1.04 (0.64–1.68)	
Tertile 3 (high)	108/85	1.78 (1.11–2.86)*		1.83 (1.07–3.13)*		1.85 (1.06–3.22)*	
**Traditional**			0.404		0.62		0.969
Tertile 1 (low)	98/92	Reference		Reference		Reference	
Tertile 2	93/99	1.09 (0.69–1.70)		1.12 (0.70–1.77)		1.16 (0.72–1.87)	
Tertile 3 (high)	95/97	1.23 (0.76–1.99)		1.14 (0.68–1.89)		1.00 (0.59–1.70)	
**Coarse cereals**			0.004		0.001		0.005
Tertile 1 (low)	87/104	Reference		Reference		Reference	
Tertile 2	96/94	1.44 (0.93–2.25)		1.54 (0.97–2.45)		1.55 (0.96–2.50)	
Tertile 3 (high)	103/90	1.95 (1.24–3.07)*		2.22 (1.37–3.60)*		2.05 (1.24–3.39)*	

^a^Cases with gastric mucosal atrophy/non-cases in tertiles.

Model 1: adjusted for age and gender.

Model 2: further adjusted for year of education, residence, marital status, occupation, income, BMI (body mass index), smoking and drinking.

Model 3: further adjusted for *H.* *pylori* infection, history of diabetes, history of hypertension, history of taking anticoagulants and total energy intake.

*p < 0.05.

The association of dietary pattern and endoscopic gastric mucosal atrophy after stratifying the participants by selected variables is shown in Supplementary Table 4. After controlling for potential confounders, The “coarse cereals” pattern was associated with endoscopic gastric mucosal atrophy in men (tertile 1 vs. tertile 3: adjusted OR = 3.05, 95% CI: 1.45–6.38, *p*-value for trend = 0.003). The “alcohol and fish” pattern was associated with *H. pylori* infection status (adjusted OR = 2.12 (95% CI: 1.08–4.15, *p*-value for trend = 0.031). The “coarse cereals” pattern was associated with endoscopic gastric mucosal atrophy in patients without *H. pylori* infection (adjusted OR = 2.35 (95% CI: 1.28–4.32, *p*-value for trend = 0.005). The results of sensitivity analysis with or without adjusting for total energy intake were similar (data not shown).

## Discussion

After adjusting for potential confounders, participants with a dietary pattern including high alcohol and fish intake had increased odds of endoscopic gastric mucosal atrophy. The “coarse cereals” pattern also increased the risk of endoscopic gastric mucosal atrophy. Vegetable, wheat, and red meat consumption were not associated with the presence of endoscopic gastric mucosal atrophy. Analysis after stratification revealed that the “alcohol and fish” pattern was associated with an increased risk for endoscopic gastric mucosal atrophy in men but not in women, which might reflect the inclusion of only five female drinkers. The “alcohol and fish” and “coarse cereals” patterns were associated with endoscopic gastric mucosal atrophy in patients without *H. pylori* infection. Although the most common cause of gastritis is *H. pylori* infection, *H. pylori-*negative gastritis was common and more likely in past alcohol drinkers than in patients with *H. pylori* infection^29^.

To the best of our knowledge, this is the first study to investigate the relationship of dietary patterns and chronic atrophic gastritis. Most previous studies investigated the association of dietary patterns and gastric cancer. A meta-analysis of 23 studies reported that diets rich in vegetables, fruit, fish, low-fat milk, and whole grains may decrease the risk of stomach cancer. Western-style diets rich in meats, refined grains, sweets, high-fat dairy products, high-fat gravies, and alcohol may increase the risk of stomach cancer^30^. Another recent meta-analysis of cohort studies found an inverse relationship between the adherence the Mediterranean Diet and gastric cancer risk^31^.

The results of previous studies of the association of diet and chronic atrophic gastritis were limited to consumption of single foods or alcohol consumption. You *et al*.^32^ revealed that alcohol was a risk factor (OR = 3.2), but Gao *et al*.^33^ found that life-time moderate beer consumption (≤51.38 g of alcohol/day) was inversely related to chronic atrophic gastritis (OR = 0.73). The majority of endoscopic and histological data do not support a relationship of alcohol consumption with chronic atrophic gastritis^34–36^. These cross-sectional studies did not find an independent association of alcohol drinking and chronic atrophic gastritis, but small sample sizes make it difficult to confirm the presence or absence of a relationship. A recent retrospective cohort study of 10,185 subjects did find that alcohol consumption was an independent risk factor of mucosal atrophy (adjusted HR = 1.001, 95% CI: 1.001–1.002)^37^. Both acute and chronic alcohol consumption can alter gastric acid secretion and induce acute gastric mucosal injury by mediating the release of inflammatory factors, granulocyte activation, protease secretion, production of reactive oxygen species, vasoconstriction, and increased vascular permeability^38,39^. A meta-analysis of cross-sectional studies found that moderate alcohol intake had a dose-response association with a reduction in *H*. *pylori* infection, but after adjusting for *H. pylori* infection status, alcohol drinking was significantly associated with gastritis in this study^40^.

There is little evidence of a direct relationship of fish consumption and chronic atrophic gastritis. A study by Tanigawa *et al*.^41^, reported that fish consumption promoted *H*. *pylori*-induced gastritis in a Mongolian gerbil model. More data are available on the effect of fish consumption on *H*. *pylori* infection. Tongtawee *et al*.^42^ reported that *H*. *pylori* infections were more frequent in those who ate pickled fish than in those who did not. Ikezaki *et al*.^43^ reported that increased fish consumption was negatively associated with the success of oral treatment to eradication of *H*. *pylori* infection. The incorporation in cell membranes of highly unsaturated n-3 fatty acids from fish could increase the risk of *H*. *pylori* gastritis because of disruption of the clustering of lipid rafts and alteration of immune responses^44,45^. Yoshinori *et al*.^46^ found that the serum concentration of docosahexaenoic acid, an n-3 polyunsaturated fatty acid, increased the risk of chronic atrophic gastritis (OR = 2.2).

A high intake of legumes and cereal grains other than wheat and rice, has long been recognized as a poor man’s dietary pattern^47^. Coarse cereals are recommended for their anti-inflammatory, anti-atherogenic, and antioxidant activities and for reducing the risk of type 2 diabetes, cardiovascular diseases, and cancer, but the effect of adherence to a “coarse cereals” diet on chronic gastritis has not been addressed^47–49^. The unfavorable effects of coarse cereals on chronic gastritis may be related to its relatively limited their nutritive value. For example some study participants routinely ate millet porridge for breakfast. The pericarp of millet is unpalatable and may contain antinutrients such as phytates and tannins^47,50^. Another possible explanation is that cereals other than yellow corn^51^, corn do not contain vitamin B_12_, vitamin C, or vitamin A, which could lead to gastritis-induced vitamin deficiency^52^ and gastric mucosal atrophy^53^. Tsigane *et al*.^3^ reported that frequent intake of soybean products was associated with an increased risk of atrophic gastritis.

This study has several strengths. First, to our knowledge, it is the first study to evaluate the association between dietary patterns and endoscopic gastric mucosal atrophy among adults in the city of Binzhou, Shandong Province, China. It provides evidence into the potential role of dietary modification in the prevention of gastric mucosal atrophy. Second, face-to-face interview ensured that the data we collected are accurate. Furthermore, we have adjusted for potential known confounders in our analyses. Nevertheless, several limitations also need to be considered. The analysis was controlled for many potential confounders, but family history of gastric cancer, physical activity, and mental health status were not considered and could have affected the results. Secondly, some participants may have had pre-existing gastric mucosal atrophy. Consequently, changing their usual dietary intake might have influenced our results. Thirdly, the cross-sectional design did not allow determining the causal relationship of dietary patterns and endoscopic gastric mucosal atrophy. Fourthly, recall bias and misclassification of study participants was not avoidable because the FGFQ had not been previously validated. Finally, because the subjects were recruited in Binzhou, China, the study findings might not be generalizable.

In conclusion, adherence to “alcohol and fish” or “coarse cereals” dietary patterns was associated with increased odds of endoscopic gastric mucosal atrophy. The results support confirmation in large prospective cohort studies.

## Supplementary information

Supplementary data
