# Supplementary material for: Association of dietary patterns and endoscopic gastric mucosal atrophy in an adult Chinese population
Source: Sci Rep. 2019 Nov 12;9:16567. doi: 10.1038/s41598-019-52951-7 (PMC6851133; doi:10.1038/s41598-019-52951-7)
Supplement: Supplementary file 1 — Supplementary data [file 41598_2019_52951_MOESM1_ESM.pdf]

Title: Association of dietary patterns and endoscopic gastric mucosal atrophy in an adult Chinese population

Authors: Song Lin, PHD[1], Tao Gao, MD[2], Chongxiu Sun, MD[1], Mengru Jia, MD[1], Chengxia Liu, PHD[2], Xingbin Ma, PHD[2], Aiguo Ma, PHD[1,*]

Affiliations: [1]The College of Public Health, Qingdao University, 38 Dengzhou Road, Qingdao, Shandong 266021, China; [2] Digestive endoscopy center, Hospital Affiliated Binzhou Medical University, Binzhou 256603, China.

***Correspondence:** Prof. Aiguo Ma, The College of Public Health, Qingdao University, 38 Dengzhou Road, Qingdao, Shandong 266021, China; Email: magfood@qdu.edu.cn; Tel: +86 532 82991518; Fax: +86 532 83812434

Supplement table 1. Definition of the food groups used in the dietary pattern analysis.

Food groups	Items
Vegetables	All kind of green leafy vegetables, tomato, cucumber, pepper, carrot, radish, eggplant, broccoli, cauliflower, and onion
Salted vegetables	Pickled garlic, cucumber, eggplant, radish, and Chinese cabbage
Fruits	Apple, orange, pear, and banana
Legumes	Soybeans, beans, and peanuts
Coarse cereals	Millet, maize, sorghum, and oats
Cooked wheaten food	Wheat
Cooked rice food	Rice
Potatoes	Potato and sweet potato
Red meat	Pork, beef, and lamb
Processed meat	Ham, sausage, and bacon
Poultry	Chicken, duck, and goose
Dairy and its product	Milk and yogurt
Beer	Bear
Wine	Wine
Sea fish	Sardine, ribbonfish, herring, butterfish, squid, and shrimp
Freshwater fish	Carp, grass carp, crucian carp, and loach
Egg	Egg
Corn oil	Corn oil
Peanut oil	Peanut oil
Soya-bean oil	Soybean oil
Lard oil	Lard oil
Tea	Tea
Sugar-sweetened beverage	Fruit juice and soft drink

Supplement table 2. Relative validity of food group intakes (g/day) estimated by the FGFQ and 24h dietary recall, and correlations between FGFQ and 24h dietary recall (*n* = 180).

Food groups	FGFQ intake		24h dietary recall		Spearman's *r*	*p*
	Mean	Median (P25–P75)	Mean	Median (P25–P75)		
Fresh Vegetables	278.9	300 (200–350)	258.4	225 (150–350)	0.26	0.0005
Salted vegetables	16.6	2 (0.5–15)	9.7	0 (0–5)	0.46	<0.001
Fruits	100.9	100 (13.3–125)	52.6	0 (0–100)	0.30	0.0001
Legumes	34.3	17.9 (6.7–50)	32.3	0 (0–20)	0.13	0.0746
Coarse cereals	22.2	3.3 (0–13.3)	20.13	0 (0–50)	0.73	<0.001
Cooked wheaten food	294.3	300 (200–400)	295.6	300 (200–400)	0.40	<0.001
Cooked rice food	70.0	35.7 (13.3–100)	49.0	0 (0–100)	0.14	0.0584
Potatoes	33.7	20.0 (6.7–51.8)	34.0	0	0.19	0.0099
Red meat	66.7	35.7 (14.2–100)	53.3	0 (0–100)	0.34	<0.001
Poultry	16.9	6.7 (2.9–16.8)	8.3	0	0.23	0.0017
Dairy and its product	120.1	16.1 (0–178.5)	8.67	0	0.20	0.0063
Alcohol	4.07	0 (0–2.1)	0.58	0	0.40	<0.001
Fish	34.3	16.8 (5.0–34.6)	21.0	0	0.33	<0.001
Egg	53.1	50 (35.7–50)	42.8	50 (0–50)	0.28	0.0001

Abbreviations: FGFQ, food group frequency questionnaire.

**Supplement Table 3.** Multivariable-adjusted odds ratios for characteristics general across tertiles of dietary patterns' scores.					
	Participants[2]	Crude OR (95%CI)	*p* for trend	Multi-adjusted ORs[3]	*p* for trend
**Age (< 50, ≥ 50 year)** [1]					
Alcohol and fish			0.308		0.768
Tertile 1 (low)	94/97	Reference		Reference	
Tertile 2	92/98	1.03 (0.69–1.54)		1.23 (0.76–1.98)	
Tertile 3 (high)	105/88	0.81 (0.54–1.21)		1.05 (0.60–1.84)	
Traditional			<0.001*		0.001*
Tertile 1 (low)	73/117	Reference		Reference	
Tertile 2	105/87	0.52 (0.34–0.78)		0.46 (0.28–0.77)	
Tertile 3 (high)	113/79	0.44 (0.29–0.66)		0.38 (0.22–0.66)	
Coarse cereals			0.011*		0.211
Tertile 1 (low)	86/105	Reference		Reference	
Tertile 2	93/97	0.85 (0.57–1.28)		0.98 (0.61–1.57)	
Tertile 3 (high)	112/81	0.59 (0.40–1.62)		0.73 (0.44–1.19)	
**Gender (women, men)**					
Alcohol and fish			<0.001*		0.121
Tertile 1 (low)	115/76	Reference		Reference	
Tertile 2	98/92	1.42 (0.95–2.13)		1.24 (0.74–2.06)	
Tertile 3 (high)	43/150	5.28 (3.38–9.25)		1.51 (0.88–2.95)	
Traditional			<0.001*		<0.001*
Tertile 1 (low)	124/66	Reference		Reference	
Tertile 2	94/98	1.96 (1.30–2.96)		1.92 (1.11–3.31)	
Tertile 3 (high)	38/154	7.61 (4.79–12.11)		5.69 (3.07–10.56)	
Coarse cereals			0.310		0.446
Tertile 1 (low)	82/109	Reference		Reference	
Tertile 2	101/89	0.66 (0.44–0.99)		0.52 (0.30–0.90)	
Tertile 3 (high)	73/120	1.24 (0.82–1.86)		0.79 (0.45–1.40)	
**Education (<6, 6–12 , >12 year)**					
Alcohol and fish			<0.001*		0.773
Tertile 1 (low)	57/113/21	Reference		Reference	
Tertile 2	45/116/29	1.38 (0.93–2.04)		1.15 (0.71–1.85)	
Tertile 3 (high)	32/107/54	2.58 (1.72–3.87)		0.89 (0.51–1.55)	
Traditional			0.021*		0.084
Tertile 1 (low)	58/98/34	Reference		Reference	
Tertile 2	44/114/34	1.28 (0.86–1.90)		0.87 (0.54–1.42)	

Tertile 3 (high)	32/124/36	1.59 (1.07–2.37)		0.62 (0.36–1.06)	
Coarse cereals			<0.001*		0.021*
Tertile 1 (low)	60/108/23	Reference		Reference	
Tertile 2	41/123/26	1.45 (0.98–2.14)		1.35 (0.84–2.16)	
Tertile 3 (high)	33/105/55	2.66 (1.77–3.99)		1.79 (1.09–2.93)	
**Residence (rural, urban)**					
Alcohol and fish			<0.001*		0.056
Tertile 1 (low)	151/40	Reference		Reference	
Tertile 2	145/45	1.17 (0.72–1.90)		0.90 (0.49–1.68)	
Tertile 3 (high)	115/78	2.56 (1.63–4.02)		1.85 (0.97–3.53)	
Traditional			0.456		0.347
Tertile 1 (low)	131/59	Reference		Reference	
Tertile 2	141/51	0.80 (0.52–1.25)		0.86 (0.48–1.53)	
Tertile 3 (high)	139/53	0.85 (0.54–1.32)		0.74 (0.40–1.38)	
Coarse cereals			0.016*		0.828
Tertile 1 (low)	145/46	Reference		Reference	
Tertile 2	141/49	1.10 (0.69–1.74)		0.87 (0.47–1.58)	
Tertile 3 (high)	125/68	1.71 (1.10–2.67)		0.93 (0.52–1.66)	
**Income (< 1000, 1000–3000, > 3000 CNY/month/per-individual)**					
Alcohol and fish			<0.001*		0.955
Tertile 1 (low)	61/69/61	Reference		Reference	
Tertile 2	41/72/77	1.55 (1.07–2.26)		0.79 (0.49–1.28)	
Tertile 3 (high)	22/53/118	3.49 (2.36–5.16)		1.10 (0.61–1.97)	
Traditional			<0.001*		0.560
Tertile 1 (low)	65/52/73	Reference		Reference	
Tertile 2	39/75/78	1.43 (0.98–2.08)		1.15 (0.71–1.86)	
Tertile 3 (high)	20/67/105	2.54 (1.73–3.74)		1.17 (0.67–2.04)	
Coarse cereals			<0.001*		0.273
Tertile 1 (low)	50/72/69	Reference		Reference	
Tertile 2	37/76/77	1.28 (0.89–1.85)		1.51 (0.93–2.43)	
Tertile 3 (high)	37/46/110	2.08 (1.42–3.06)		1.30 (0.78–2.19)	
**Body mass index (< 25, ≥ 25 kg/m^2)**					
Alcohol and fish			0.020*		0.811
Tertile 1 (low)	138/53	Reference		Reference	
Tertile 2	132/58	1.14 (0.74–1.78)		0.99 (0.61–1.62)	
Tertile 3 (high)	118/75	1.65 (1.08–2.54)		0.93 (0.54–1.61)	
Traditional			0.714		0.397
Tertile 1 (low)	123/67	Reference		Reference	
Tertile 2	144/48	0.61 (0.39–0.95)		0.56 (0.34–0.92)	

Tertile 3 (high)	121/71	1.08 (0.71–1.64)		0.80 (0.47–1.34)	
Coarse cereals			0.883		0.853
Tertile 1 (low)	126/65	Reference		Reference	
Tertile 2	136/54	0.77 (0.50–1.19)		0.85 (0.53–1.38)	
Tertile 3 (high)	126/67	1.03 (0.68–1.57)		1.05 (0.65–1.69)	
**Smoking (no, yes)**					
Alcohol and fish			<0.001*		0.076
Tertile 1 (low)	117/74	Reference		Reference	
Tertile 2	120/70	0.92 (0.61–1.40)		0.88 (0.56–1.37)	
Tertile 3 (high)	79/114	2.28 (1.52–3.43)		1.63 (0.99–2.69)	
Traditional			<0.001*		0.076
Tertile 1 (low)	121/69	Reference		Reference	
Tertile 2	108/84	1.36 (0.90–2.06)		1.25 (0.79–1.96)	
Tertile 3 (high)	87/105	2.12 (1.40–3.19)		1.51 (0.93–2.45)	
Coarse cereals			0.050*		0.046*
Tertile 1 (low)	111/80	Reference		Reference	
Tertile 2	112/78	0.97 (0.64–1.45)		1.04 (0.67–1.62)	
Tertile 3 (high)	93/100	1.49 (0.98–2.23)		1.58 (1.01–2.48)	
**Marital status (single/divorced/widowed, married)**					
Alcohol and fish			0.986		0.991
Tertile 1 (low)	6/185	Reference		Reference	
Tertile 2	4/186	1.51 (0.42–5.43)		0.91 (0.072–11.42)	
Tertile 3 (high)	6/187	1.01 (0.32–3.19)		0.96 (0.075–12.30)	
Traditional			0.743		0.239
Tertile 1 (low)	6/184	Reference		Reference	
Tertile 2	5/187	1.22 (0.37–4.07)		10.06 (0.86–118.18)	
Tertile 3 (high)	5/187	1.22 (0.36–4.07)		4.13 (0.57–36.71)	
Coarse cereals			0.073		0.474
Tertile 1 (low)	3/188	Reference		Reference	
Tertile 2	4/186	0.74 (0.16–3.36)		16.07 (0.43–595.9)	
Tertile 3 (high)	9/184	0.33 (0.87–1.22)		2.89 (0.34–24.55)	
**Drinking (no, yes)**					
Alcohol and fish			<0.001*		<0.001*
Tertile 1 (low)	178/13	Reference		Reference	
Tertile 2	164/26	2.17 (1.09–4.37)		2.17 (0.97–4.88)	
Tertile 3 (high)	83/110	18.14 (9.65–34.11)		13.07 (6.18–27.67)	
Traditional			<0.001*		0.001*
Tertile 1 (low)	168/22	Reference		Reference	
Tertile 2	150/42	2.14 (1.22–3.75)		1.85 (0.91–3.75)	

Tertile 3 (high)	107/85	6.01 (3.58–10.28)		3.08 (1.56–6.09)	
Coarse cereals			0.524		0.311
Tertile 1 (low)	139/52	Reference		Reference	
Tertile 2	151/39	0.69 (0.43–1.10)		0.89 (0.49–1.62)	
Tertile 3 (high)	135/58	1.15 (0.74–1.79)		0.74 (0.42–1.32)	
***H. pylori* infection (no, yes)**					
Alcohol and fish			0.910		0.482
Tertile 1 (low)	132/59	Reference		Reference	
Tertile 2	143/45	0.70 (0.45–1.11)		0.68 (0.42–1.09)	
Tertile 3 (high)	131/60	1.02 (0.66–1.58)		0.86 (0.51–1.46)	
Traditional			0.137		0.241
Tertile 1 (low)	137/51	Reference		Reference	
Tertile 2	143/48	0.90 (0.57–1.42)		0.88 (0.54–1.42)	
Tertile 3 (high)	126/65	1.39 (0.89–2.15)		1.34 (0.81–2.23)	
Coarse cereals			0.021*		0.016*
Tertile 1 (low)	144/46	Reference		Reference	
Tertile 2	137/51	1.17 (0.73–1.85)		1.18 (0.73–1.90)	
Tertile 3 (high)	125/67	1.68 (1.07–2.62)		1.78 (1.11–2.86)	
**History of diabetes (no, yes)**					
Alcohol and fish			0.075		0.047
Tertile 1 (low)	13/178	Reference		Reference	
Tertile 2	14/176	0.92 (0.42–2.01)		1.07 (0.43–2.67)	
Tertile 3 (high)	5/188	2.75 (0.96–7.86)		4.52 (1.18–17.33)	
Traditional			0.027*		0.403
Tertile 1 (low)	15/175	Reference		Reference	
Tertile 2	12/180	1.29 (0.59–2.82)		0.88 (0.35–2.18)	
Tertile 3 (high)	5/187	3.21 (1.14–9.01)		1.88 (0.55–6.38)	
Coarse cereals			0.521		0.557
Tertile 1 (low)	10/181	Reference		Reference	
Tertile 2	9/181	1.11 (0.44–2.80)		1.22 (0.44–3.41)	
Tertile 3 (high)	13/180	0.76 (0.33–1.79)		0.75 (0.28–1.98)	
**History of hypertension (no, yes)**					
Alcohol and fish			0.023*		0.019*
Tertile 1 (low)	15/176	Reference		Reference	
Tertile 2	26/164	0.54 (0.28–1.05)		0.41 (0.19–0.90)	
Tertile 3 (high)	30/163	0.46 (0.24–0.89)		0.37 (0.15–0.88)	
Traditional			0.058		0.122
Tertile 1 (low)	32/158	Reference		Reference	
Tertile 2	19/173	1.84 (1.00–3.38)		1.80 (0.86–3.79)	

Tertile 3 (high)	20/172	1.74 (0.96–3.17)		1.81 (0.82–4.01)	
Coarse cereals			0.231		0.176
Tertile 1 (low)	21/170	Reference		Reference	
Tertile 2	21/169	0.99 (0.52–1.89)		0.76 (0.37–1.57)	
Tertile 3 (high)	29/164	0.70 (0.38–1.27)		0.61 (0.29–1.25)	
**History of taking anticoagulants (no, yes)**					
Alcohol and fish			0.048*		0.415
Tertile 1 (low)	7/184	Reference		Reference	
Tertile 2	8/182	0.87 (0.31–2.43)		0.91 (0.29–2.84)	
Tertile 3 (high)	16/177	0.42 (0.17–1.05)		0.63 (0.20–1.97)	
Traditional			0.669		0.694
Tertile 1 (low)	10/180	Reference		Reference	
Tertile 2	9/183	1.13 (0.45–2.85)		1.26 (0.44–3.55)	
Tertile 3 (high)	12/180	0.83 (0.35–1.98)		1.25 (0.43–3.61)	
Coarse cereals			0.251		0.260
Tertile 1 (low)	14/177	Reference		Reference	
Tertile 2	8/182	1.80 (0.74–4.39)		1.57 (0.59–4.20)	
Tertile 3 (high)	9/184	1.62 (0.68–3.83)		1.71 (0.65–4.53)	
**Occupation (not farmer, farmer)**					
Alcohol and fish			<0.001*		0.020*
Tertile 1 (low)	89/102	Reference		Reference	
Tertile 2	115/75	0.57 (0.38–0.85)		0.48 (0.26–0.88)	
Tertile 3 (high)	136/57	0.37 (0.24–0.56)		0.46 (0.22–0.99)	
Traditional			0.182		0.571
Tertile 1 (low)	107/83	Reference		Reference	
Tertile 2	112/80	0.92 (0.61–1.38)		1.02 (0.54–1.92)	
Tertile 3 (high)	121/71	0.76 (0.50–1.13)		1.25 (0.60–2.63)	
Coarse cereals			<0.001*		0.207
Tertile 1 (low)	94/97	Reference		Reference	
Tertile 2	109/81	0.72 (0.48–1.08)		0.86 (0.47–1.57)	
Tertile 3 (high)	137/56	0.40 (0.26–0.60)		0.65 (0.34–1.26)	

Abbreviations: OR, odd ratio; CNY, Chinese yuan.

[1] First group is reference (e.g. age < 50 year as reference group).

[2] Number of each group's participants in tertiles (starting from the reference group).

[3] Except for the change of independent variable, models adjusted for as model 3 in Table 4. In addition, the association between adherence to each dietary pattern and education, income was tested by ordered logistic regression in different models.

*$p < 0.05$.

Supplement table 4. Multivariable-adjusted odds ratios for endoscopic gastric mucosal atrophy across tertiles of dietary patterns' scores, stratified by gender and *H. pylori* infection status.

	Cases/non-cases[1]	Model 1	*p* for trend	Model 2	*p* for trend	Model 3	*p* for trend
**Women**							
Alcohol and fish			0.041*		0.046*		0.104
Tertile 1 (low)	51/64	Reference		Reference		Reference	
Tertile 2	46/52	1.21 (0.68-2.15)		1.24 (0.68-2.26)		1.16 (0.63-2.16)	
Tertile 3 (high)	27/16	2.34 (1.09-5.05)		2.40 (1.06-5.43)		2.12 (0.92-4.89)	
Traditional			0.393		0.332		0.354
Tertile 1 (low)	62/62	Reference		Reference		Reference	
Tertile 2	41/53	0.94 (0.53-1.70)		1.01 (0.56-1.85)		1.16 (0.62-2.17)	
Tertile 3 (high)	21/17	1.58 (0.72-3.45)		1.69 (0.71-4.02)		1.52 (0.62-3.74)	
Coarse cereals			0.221		0.179		0.334
Tertile 1 (low)	37/45	Reference		Reference		Reference	
Tertile 2	49/52	1.28 (0.69-2.38)		1.48 (0.76-2.88)		1.37 (0.69-2.74)	
Tertile 3 (high)	38/35	1.52 (0.77-3.00)		1.64 (0.79-3.41)		1.45 (0.68-3.09)	
**Men**							
Alcohol and fish			0.166		0.350		0.132
Tertile 1 (low)	40/36	Reference		Reference		Reference	
Tertile 2	41/51	0.63 (0.31-1.28)		0.60 (0.27-1.32)		0.74 (0.32-1.74)	
Tertile 3 (high)	81/69	1.38 (0.72-2.62)		1.34 (0.60-3.02)		1.79 (0.75-4.26)	
Traditional			0.706		0.682		0.333
Tertile 1 (low)	36/30	Reference		Reference		Reference	
Tertile 2	52/46	1.24 (0.61-2.59)		1.23 (0.58-2.64)		1.15 (0.50-2.62)	
Tertile 3 (high)	74/80	1.18 (0.61-2.30)		0.92 (0.44-1.93)		0.74 (0.34-1.65)	

Coarse cereals			0.004*		0.001*		0.003*
Tertile 1 (low)	50/59	Reference		Reference		Reference	
Tertile 2	47/42	1.58 (0.84-3.00)		1.80 (0.91-3.56)		1.85 (0.89-3.82)	
Tertile 3 (high)	65/55	2.50 (1.34-4.69)		3.03 (1.53-6.03)		3.05 (1.45-6.38)	
**Negative *H. pylori* infection**							
Alcohol and fish			0.010*		0.022*		0.031*
Tertile 1 (low)	51/81	Reference		Reference		Reference	
Tertile 2	59/84	1.29 (0.75-2.20)		1.33 (0.76-2.33)		1.30 (0.74-2.29)	
Tertile 3 (high)	65/66	2.19 (1.21-3.97)		2.19 (1.13-4.24)		2.12 (1.08-4.15)	
Traditional			0.924		0.720		0.789
Tertile 1 (low)	64/73	Reference		Reference		Reference	
Tertile 2	63/80	1.24 (0.73-2.11)		1.25 (0.72-2.18)		1.31 (0.75-2.30)	
Tertile 3 (high)	48/78	1.10 (0.56-1.83)		0.86 (0.45-1.63)		0.88 (0.46-1.69)	
Coarse cereals			0.016*		0.005*		0.005*
Tertile 1 (low)	56/88	Reference		Reference		Reference	
Tertile 2	62/75	1.59 (0.94-2.68)		1.72 (1.00-2.98)		1.74 (1.00-3.02)	
Tertile 3 (high)	57/68	1.95 (1.12-3.39)		2.34 (1.28-4.26)		2.35 (1.28-4.32)	
**Positive *H. pylori* infection**							
Alcohol and fish			0.520		0.293		0.285
Tertile 1 (low)	40/19	Reference		Reference		Reference	
Tertile 2	27/18	0.58 (0.23-1.47)		0.56 (0.19-1.66)		0.51 (0.16-1.62)	
Tertile 3 (high)	42/18	1.38 (0.55-3.43)		2.11 (0.61-7.29)		2.19 (0.61-7.86)	
Traditional			0.830		0.497		0.607
Tertile 1 (low)	34/17	Reference		Reference		Reference	
Tertile 2	29/19	0.65 (0.25-		0.57 (0.19-		0.46 (0.14-	

		1.70)		1.75)		1.50)	
Tertile 3 (high)	46/19	1.08 (0.41-2.86)		1.41 (0.46-4.38)		1.26 (0.39-4.06)	
Coarse cereals			0.501		0.174		0.119
Tertile 1 (low)	31/15	Reference		Reference		Reference	
Tertile 2	33/18	0.96 (0.37-2.48)		1.28 (0.42-3.91)		1.40 (0.43-4.49)	
Tertile 3 (high)	45/22	1.35 (0.54-3.37)		2.10 (0.69-6.35)		2.48 (0.76-8.05)	

[1] Cases with gastric mucosal atrophy/non-cases in tertiles.

In gender subgroups, model 1 only adjusted for age. Other models adjusted for as in Table 4.

*$p < 0.05$.
